# Efficacy of Iranian Traditional Medicine in the Treatment of Epilepsy

**DOI:** 10.1155/2013/692751

**Published:** 2013-07-07

**Authors:** Mehri Abdollahi Fard, Asie Shojaii

**Affiliations:** Research Institute for Islamic and Complementary Medicine, Iran University of Medical Sciences, Jomhuri Avenue, P.O. Box 1145847111, Tehran, Iran

## Abstract

Epilepsy is a brain disorder which affects about 50 million people worldwide. Ineffectiveness of the drugs in some cases and the serious side effects and chronic toxicity of the antiepileptic drugs lead to use of herbal medicine as a form of complementary and alternative medicine. In this review modern evidences for the efficacy of antiepileptic medicinal plants in Traditional Iranian Medicine (TIM) will be discussed. For this purpose electronic databases including PubMed, Scopus, Sciencedirect, and Google Scholar were searched for each of the antiepileptic plants during 1970-February 2013.Anticonvulsant effect of some of the medicinal plants mentioned in TIM like *Anacyclus pyrethrum, Pimpinella anisum, Nigella sativa,* and *Ferula gummosa* was studied with different models of seizure. Also for some of these plants like *Nigella sativa* or *Piper longum* the active constituent responsible for antiepileptic effect was isolated and studied. For some of the herbal medicine used in TIM such as *Pistacia lentiscus* gum (Mastaki), *Bryonia alba* (Fashra), *Ferula persica* (Sakbinaj), *Ecballium elaterium* (Ghesa-al Hemar), and *Alpinia officinarum* (Kholanjan) there is no or not enough studies to confirm their effectiveness in epilepsy. It is suggested that an evaluation of the effects of these plants on different epileptic models should be performed.

## 1. Introduction

Epilepsy is a brain disorder which is characterized by occurrence of spontaneous seizures induced by a complex of neurotransmitter systems. It is one of the most common neurological disorders which affects about 50 million people worldwide [1]. It is estimated that about 1% of people through the word suffer from epilepsy [2]. Epilepsy can commonly be controlled with modern anticonvulsants which prevent the seizures or reduce their intensity. However, over 30% of people with epilepsy have uncontrolled seizures even with the best available drugs, but there is some difficulty in the treatment procedure of epilepsy, for example, ineffectiveness of the drugs in nearly 20% of the cases and the serious side effects and chronic toxicity of the drugs [3]. Therefore there is an increasing interest to use natural sources which would be a valuable agent for finding the new therapeutic drugs. On the other hand, the World Health Organization has estimated that perhaps 80% of the world's population relies chiefly on traditional medicine for primary health care needs. Although, most of the herbal and complementary medicines are used without adequate evidence of benefit and safety from controlled clinical trials.

The ancient Iranian medicine was combined by different medical traditions from Greece, Egypt, India, and China for more than 4000 years. TIM consists of the sum total of all the knowledge and practices used in diagnosis, prevention, and elimination in Persia from ancient times to present. There is information and data in Traditional Iranian documents or books about medicinal herbs which are used by TIM scientists for the treatment of epilepsy [4]. 

In this study, we reviewed the herbal treatments for epilepsy mentioned by TIM documents and text and compared them with the recent scientific literature. 

## 2. Materials and Methods

In this paper, we reviewed the most important ancient Herbal treatments for epilepsy mentioned by the most famous TIM books like Makhzan, Tohfeh, Tebe Akbari, and Moalejate Aghili, and considered this medicinal herbs as the key words for the next step of our study [5–8]. Then electronic databases including PubMed, Scopus, ScienceDirect, and Google Scholar were searched for each of the plants in TIM plus epilepsy, anticonvulsant, or seizure as the keywords. These results revealed new scientific findings in modern medicine about the TIM recommendations for epilepsy in order to design future scientific studies based on the suggested herbal therapies for epilepsy.

## 3. Results

There are some medicinal plants which were used in TIM for the treatment of epilepsy. The results showed that there are scientific evidences for antiepileptic effects of some of these plants in the literature (Table 1).

### 3.1. Modern Evidence for the Efficacy of Medicinal Plants in TIM Used for the Treatment of Epilepsy

#### 3.1.1. *Terminalia chebula*

“Halile siah” is the black fruit of *Terminalia chebula* which has been used in TIM for the treatment of epilepsy. The anticonvulsant activity of the ethanolic extract of *Terminalia chebula* (200 and 500 mg/kg p.o.) was investigated in albino mice by the use of maximum electroshock seizure (MES) test, pentylenetetrazole (PTZ), and picrotoxin test.

The findings of the study revealed that the ethanolic extract of *Terminalia chebula* significantly reduced the duration of seizures induced by MES. The ethanol extract in doses of 200 and 500 mg/kg conferred protection (17 and 50%, resp.) on the mice. The same doses also protected animals from PTZ-induced tonic seizures and significantly delayed the onset of tonic seizures produced by picrotoxin. So the ethanolic extract of *Terminalia chebula* possesses anticonvulsant activity in mice [9].

#### 3.1.2. *Pimpinella anisum*

Anisun is a traditional name of *Pimpinella anisum* seeds that has been used for the treatment of epilepsy in TIM. The seeds (fruits) of this plant have been studied for anticonvulsant activity. The effects of an essential oil of the fruits of *Pimpinella anisum* against seizures induced by PTZ or MES were investigated in male mice. The essential oil suppressed hind limb tonic extensions (HLTE) and mortality in PTZ-induced seizure (ED_50_ = 0.52 mL/Kg) and MES-induced seizure (ED_50_ = 0.2 mL/Kg). It also elevated the threshold of PTZ-induced clonic convulsions in mice [14]. Also the methanolic extract of the anise seeds in doses of 12.5–400 mg/Kg was investigated for anticonvulsant activity in mice using Picrotoxin model. The results revealed that the extract showed a significant anticonvulsant activity in a dose of 200 mg/Kg compared to the control groups [15]. In another study antiseizure and antihypoxia effects of anise oil were studied on PTZ model and neuronal hypoxia induced by oxygen withdrawal as well as on the production of dark neurons and induction of long-term potentiation (LTP) in vivo and in vitro experimental models of rat brain. Anise oil significantly prolonged the latency of seizure attacks and reduced the amplitude and duration of epileptiform burst discharges induced by the injection of PTZ. In addition, anise oil significantly inhibited the production of dark neurons in different regions of the brain in epileptic rats. Anise oil also significantly enhanced the duration of the appearance of anoxic terminal negativity induced by oxygen withdrawal and inhibited the induction of LTP in hippocampal slices. So this study indicates anticonvulsant and neuroprotective effects of anise oil [16, 17].

#### 3.1.3. *Anacyclus pyrehrun*

*Anacyclus pyrethrum*, known as “Agher Gherha” in TIM, is another drug used in TIM for the treatment of epilepsy and seizure. In a study by Zaidi et al. anticonvulsant, antioxidant, and neuropharmacological activities of the chloroform fraction of *Anacyclus pyrethrum* roots at a dose range of 100–800 mg/kg (i.p.) were evaluated in mice. PTZ and increasing current electroshock (ICES) models were used. The chloroform fraction significantly (*P* < 0.001) delayed the onset of PTZ-induced seizures. ICES test results significantly increased the seizure threshold. This fraction also causes a reduction in the formation of lipid peroxidation products but did not show antianxiety and antidepressant effects. The results indicate potential antiseizure activity of chloroform fraction of *Anacyclus pyrethrum* root extract along with additional neuropharmacological effects [10].

The anticonvulsant and myorelaxation activities of *A. pyrethrum* root ethanolic extract were evaluated in albino mice using MES model and rotarad test, respectively. The extract was prescribed in doses of 200, 400, and 600 mg/kg i.p. and compared with control groups (group I, vehicle treated, group II, phenytoin treated). The extract of *A. pyrethrum* reduced the duration of hind limb tonic extension in a dose-dependent manner in MES model [11]. In another study the effect of hydroalcoholic extract of *A. pyrethrum* root extract was studied against seizures, seizure-induced oxidative stress, and cognitive impairment in male Wistar rats. The extract was administered in doses of 50, 100, 250, and 500 in PTZ model and 250, 500, and 1000 mg/kg in MES model. Myoclonic jerk latency and generalized tonic clonic seizures (GTCS) were noted in PTZ whereas occurrence of tonic hind limb extension (THLE) was observed in MES seizures. Cognitive deficit was assessed using the elevated plus maze and passive avoidance tests. Whole brain reduced glutathione, malondialdehyde levels and cholinesterase activity were measured. In the PTZ-induced seizures, *A. pyrethrum* root extract showed a 50, 66.7, 83.3, and 100% protection against GTCS at doses 50, 100, 250, and 500 mg/kg, respectively. In MES model, the extract produced a 16.7, 33.3, and 50% protection against THLE at doses of 250, 500, and 1000 mg/kg, respectively. Root extract also significantly prevented seizure-induced oxidative stress and cognitive impairment in a dose-dependent manner [12]. Recently Pahuja et al. evaluated the effect of orally administration of hydroalcoholic extracts of *A. pyrethrum* root in the doses of 100, 250 and 500 mg/kg in the PTZ-induced kindling, spatial memory, oxidative stress, and rho kinase (ROCK II) in albino mice. Pretreatment with the extract at doses of 250 and 500 mg/kg showed significant increase in the myoclonic jerk latency and delay in the development of kindling. A significant decrease in mortality was observed at higher doses of the extract (250 and 500 mg/kg). Pretreatment with the root extract significantly increased the number of platform crossings and decreased the escape latency, as opposed to the PTZ group, thus showing protection against the memory deficit. The oxidative stress induced by PTZ kindling is also attenuated. PTZ-induced kindling increased the ROCK II expression whereas pretreatment with *Anacyclus* extract attenuated the increase in ROCK II expression [13].

#### 3.1.4. *Laurus nobilis*

*Laurus nobilis* (Ghar in TIM), Lauraceae, is a plant which is mentioned for antiepileptic effects in the ancient books of Iran. The leaf's essential oil of *Laurus nobilis* Linn was evaluated for anticonvulsant activity against experimental seizures. The essential oil protected mice against tonic convulsions induced by maximal electroshock and especially by PTZ (ED_50_ = 0.19 mL/Kg). Components responsible for this effect may be methyleugenol, eugenol, and pinene present in the essential oil. At anticonvulsant doses, the essential oil produced sedation and motor impairment. This effect seems to be related in part to cineol, eugenol, and methyleugenol. Although the essential oil had an acceptable acute toxicity, further studies are required before any absolute conclusions can be drawn [18].

#### 3.1.5. *Origanum majorana*

The aerial parts of *Origanum majorana* (“Marzanjoosh” in TIM) are used for the treatment of epilepsy and some CNS disorders in TIM.

Anticonvulsant and sedative activities for different extracts of aerial parts (leaves and stems) of *O. majorana* were investigated using the PTZ and maximal electroshock (MES) test. Different extracts of *O. majorana* including pet. ether, chloroform, acetone, methanol, and aqueous extracts exhibited an anticonvulsant effect in both the PTZ and MES-induced seizure models at the doses of 250 and 500 mg/kg i.p. The extracts of *O. majorana* delayed the onset of seizures and reduced the duration of seizures in PTZ test and decreased the duration of seizures in MES test compared to the control group. The chloroform extract exhibited a maximum reduction (58.47 and 44.83% in PTZ and MES test, resp.) in the duration of seizures. A triterpenoic acid fraction (TAF) which contained a substantial amount of ursolic acid was isolated from the chloroformic fraction. The TAF exhibited a maximum reduction (64.54 and 59.31% in PTZ and MES test, resp.) in the duration of seizures compared to the other extracts of *O. majorana*. Also, the test extracts decreased the latency and increased the duration of total sleeping time significantly. Presence of flavonoids, steroids, triterpenoids, and essential oil may be responsible for the anticonvulsant activity of this plant [19].

#### 3.1.6. *Nigella sativa*

The seeds of *Nigella sativa*, called “Shoneez” in TIM, were used in some of the herbal preparations for the treatment of epilepsy. The anticonvulsant effects of the aqueous seed extract of *Nigella sativa* were evaluated using pentylenetetrazole (PTZ—40 mg/kg b.w.) induced seizure model on adult albino rats. The effects of the extract on motor behaviour, sleeping time, seizure parameters (ictal phase duration, interictal phase duration, and seizure score), and EEG were studied. The results indicated that *Nigella* extract impaired motor coordination, reduced locomotor activity but increased sleeping time. It also showed the animals with prior treatment with *Nigella* extract to have much resistance to convulsion than the control animals. Ictal phase duration and severity score decreased whereas interictal phase duration and duration of onset of seizure increased in *Nigella* treated group. In EEG recording spike/wave and burst discharges were reduced considerably after *Nigella* treatment. Picrotoxin, a GABAA antagonist, antagonized the reduction of seizure activity by *N. sativa* inhibited the prolongation of seizure latency. The findings of this study suggest that *N. sativa* may have an anticonvulsant activity in the petit mal epilepsy probably through an increase in GABAergic tone [20]. Also the anticonvulsant and antioxidant activities of *Nigella* seed oil on PTZ-kindling seizures in mice were investigated compare to valproate. Both *Nigella* oil and valproate significantly decreased the oxidative injury in the mouse brain tissue in comparison with the PTZ-kindling group. *Nigella sativa* oil was found to be the most effective in preventing PTZ-induced seizures relative to valproate. *Nigella sativa* oil showed antiepileptogenic properties as it reduced the sensitivity of kindled mice to the convulsive and lethal effects of PTZ; valproate was ineffective in preventing the development of any of these effects [21]. Akhondian et al. evaluated the efficacy of the aqueous extract of *Nigella sativa* in reducing the frequency of seizures in childhood refractory epilepsy. This study was a double-blinded crossover clinical trial conducted on children with refractory epilepsy. The extract was administered as an adjunct therapy, and the effects were compared with those of a placebo. All the patients (20 children, 13 months to 13 years old) were receiving constant treatment for at least one month before the study. They received extract (40 mg/kg/8 h) or placebo for a period of four weeks, and between these periods for two weeks they received only their preexisting antiepileptic drugs (AEDs). The mean frequency of seizures decreased significantly during the treatment with the extract (*P* < 0.05) so the water extract of *Nigella sativa *L. has antiepileptic effects in children with refractory seizures [22]. 

Thymoquinone is the major constituent of *Nigella sativa* seeds, and anticonvulsant activity of this compound was evaluated in experimental and clinical studies. 

Anticonvulsant activity of thymoquinone was investigated in PTZ and MES models of seizure in dose of 40, 80 mg/kg prolong the onset of seizures and reduce the duration of myoclonic seizures induced by PTZ treatment but not by MES. But the complete protective effect against mortality was reported in both tests. In addition this study suggests that the effect of thymoquinone in PTZ model probably through an opioid receptor mediated an increase in the GABAergic tone (base on flumazenil and naloxone response) [23]. Also the anticonvulsant effect of thymoquinone was evaluated through intracerebroventricular (i.c.v.) injection using PTZ-induced seizure model. The i.c.v. injection of thymoquinone (200 and 400 *μ*mol) prolonged the onset and reduced the duration of tonic-clonic seizures. The protective effect of thymoquinone against lethality was 45% and 50% in the mentioned doses, respectively. In this study, flumazenil (1 nmol, i.c.v.) reversed the anticonvulsant activity of thymoquinone. Also, pretreatment with naloxone (10 *μ*mol, i.c.v.) antagonized the prolongation of tonic-clonic seizure latency, as well as reduction in seizure duration both induced by thymoquinone (200 *μ*mol, i.c.v.). So thymoquinone may possess anticonvulsant activity probably through an opioid receptor-mediated increase in GABAergic tone [24].

A pilot, double-blinded crossover clinical trial study of thymoquinone (1 mg/kg) as an adjunctive therapy was performed on children with refractory epilepsy, and its effects on frequency of seizures were compared with those of a placebo. The patients (22 children) were assigned in two groups and received either thymoquinone or placebo for a period of four weeks, and then, during the two weeks of wash outperiod, they received only their preexisting antiepileptic drugs; then, after cross-overing, they received thymoquinone or placebo for a period of four weeks again. During these periods their effects on seizure frequency were investigated. The reduction of frequency of seizures at the end of first period in comparison with the same period before the study demonstrated a significant difference between two groups (thymoquinone and placebo) (*P* = 0.04). Also the reduction of the frequency of seizure has shown a significant difference between the two groups at the end of second period in comparison with the end of the first period (*P* = 0.02) [25].

#### 3.1.7. *Caesalpinia bonducella* (L.)

*Caesalpinia bonducella* (L.) Fleming (Syn. Caesalpinia bonduc (L.) Roxb), known as “Bandogh Hendi” in TIM, belonging to the family Caesalpiniaceae, is widely distributed all over the world. Anticonvulsive effect of the seed petroleum ether extract of *Caesalpinia bonducella was studied using *PTZ, MES, and strychnine- and picrotoxin-induced convulsions models. Diazepam was used as a standard reference for all models except maximal electroshock model, wherein phenytoin was used as standard reference. Seed kernels of *C. bonducella *were extracted with solvents like petroleum ether, ethanol, methanol, and water using soxhlet apparatus. All the extracts were administered as a suspension in 2% gum acacia in all the experiments. In PTZ, MES, and strychnine- and picrotoxin-induced convulsion models medium and high doses (600 and 800 mg/kg) of the petroleum ether extract showed a significant anticonvulsant activity. The present study revealed that the petroleum ether extract possessed anticonvulsant activity which may be contributed to the presence of phytoconstituents such as saponins, proteins, homoisoflavone (bonducillin), carbohydrates, and sterols present in the drug, as these are already reported for their anxiolytic and anticonvulsant activities [35].

#### 3.1.8. *Piper longum*

*Piper longum* known as “Darfelfel” in TIM. The fruits of *Piper longum* were used in the treatment of epilepsy.

An aqueous extract of fruits of *Piper longum* L. PL was investigated for its anticonvulsant activity against seizures induced by PTZ, strychnine, and 4-aminopyridine in mice. The results revealed that the treatment of groups with the extract (250 mg/kg and 500 mg/kg p.o.) offered protection against PTZ-induced convulsions but failed to protect against strychnine and 4-aminopyridine-induced convulsions in mice. Diminution in gamma amino butyric acid (GABA) levels in the brain of extract the treated mice compared to vehicle the treated mice clearly indicated the involvement of GABAergic mechanisms in the anticonvulsant activity [26].

Piperine, an alkaloid present in the *Piper* genus, was shown to have an anticonvulsant activity, evaluated by the pilocarpine-induced model, in mice. Pilocarpine (350 mg/kg i.p.) was administered 30 min after piperine (2.5, 5, 10, and 20 mg/kg i.p.) which significantly increased latencies to 1st convulsion and to death and percentage of survivals. These parameters were also increased in the pilocarpine groups pretreated with atropine plus piperine (10 and 2.5 mg/kg, resp.), as related to the pilocarpine group. Moreover, the pilocarpine group pretreated with diazepam (which binds to the GABAA receptor, 0.2 and 0.5 mg/kg, i.p.) plus piperine (1 and 2.5 mg/kg) significantly increased latency to the 1st convulsion, as related to the pilocarpine group, suggesting that the GABAergic system is involved with the piperine action. Furthermore, the piperine effect was blocked by flumazenil (2 mg/kg, i.p.), a benzodiazepine antagonist. Untreated P350 animals showed decreased striatal DA and increased DOPAC and HVA levels that were not affected in the piperine plus pilocarpine groups. Piperine increased striatal levels of GABA, glycine and taurine, and reversed pilocarpine-induced increases in nitrite contents in sera and brain. Hippocampi from the untreated pilocarpine group showed an increased number of TNF-*α* immunostained cells in all areas, as opposed to the pilocarpine group pretreated with piperine. Taken together, piperine anticonvulsant effects are the result of its anti-inflammatory and antioxidant actions, as well as TNF-*α* reduction. In addition, piperine effects on inhibitory amino acids and on the GABAergic system may certainly contribute to the drug's anticonvulsant activity [28].

Also piperine has been reported to enhance the oral bioavailability of phenytoin in human volunteers. The effect of a single dose of piperine in patients with uncontrolled epilepsy on the steady-state pharmacokinetics of phenytoin was evaluated. Two groups of 10 patients each receiving either a 150 mg or 200 mg twice daily dose of phenytoin were selected. Twelve hours after the night dose, venous blood samples were collected at 0, 0.5, 1, 2, 4, 6, 9, and 12 h after administration of phenytoin. On the next study day, piperine 20 mg was administered along with phenytoin, and samples were collected similarly. The mean plasma drug concentrations at different time points and the pharmacokinetic parameters before and after piperine administration were compared by Student's *t*-test. Piperine increased significantly the mean plasma concentration of phenytoin at most of the time points in both dose groups. There was a significant increase in AUC_(0-12 h)_ (*P* < 0.01), *C*_max⁡_ (Maximum plasma concentrations) (*P* < 0.001) and *K*_*a*_ (The absorption rate Constant) (*P* < 0.05) whereas the changes in *K*_el_ (The terminal phase rate constant) and *t*_max⁡_ (the time to reach the maximum concentration) were not significant. The results showed that piperine enhanced the bioavailability of phenytoin significantly, possibly by increasing the absorption [27]. In another study the effects of piperine on convulsions induced in mice by agonists at different excitatory amino acid receptor subtypes were studied. Piperine was shown to significantly block convulsions induced by intracerebroventricular injection of threshold doses of kainate but to have no or only slight effects on convulsions induced by L-glutamate, N-methyl-D-aspartate, or guanidinosuccinate. Piperine suspensions, injected intraperitoneally, 1 h before the injection of the threshold intracerebroventricular dose of kainate for the induction of clonic convulsions (1 nmol), blocked these convulsions with an ED_50_ (and 95% confidence interval) of 46 (25–86) mg/kg [29].

#### 3.1.9. *Ferula gummosa*

“Barijeh” (Traditional name of *Ferula gummosa*) is a plant which has been mentioned for antiepileptic properties in TIM. The fruit essential oil of *Ferula gummosa*, Umbelliferae, was evaluated for anticonvulsant activity against experimental seizures induced by MES but it had no effect against seizures. However, it protected mice against PTZ-induced tonic seizures. The protective dose produced neurotoxicity. Moreover, this dose was too close to the LD_50_ of the essential oil [30]. In another study the seed acetone extract of *F. gummosa* protected mice against tonic convulsions induced by maximal electroshock (ED_50_ = 198.3 mg/kg) and especially by pentylenetetrazole (ED_50_ = 55 mg/kg). Neurotoxicity (sedation and motor impairment) of the extract was assessed by the rotarod test, and the median toxic dose (TD_50_) value of 375.8 mg/kg was obtained [31]. Also anticonvulsant effect of the root acetone extract of *F. gummosa* (300–500 mg/Kg i.p.) was evaluated in PTZ and MES models of seizure. The extract showed dose-dependent prevention of tonic seizures induced by PTZ (ED_50_ = 154.4 mg/kg. *Ferula* root extract also showed a significant protective effect against MES-induced seizures at doses of 500 and 750 mg/kg, and the higher doses were toxic) [32].

#### 3.1.10. *Ferula Assa-Foetida*

*Ferula assafoetida *known as “Anghose” in TIM is another herbal drug for treatment of epilepsy. The anticonvulsant effect of *F. assa foethica* gum ethanolic extract was investigated using PTZ-induced kindling method in male mice. The animals were divided into 6 experimental groups (*n* = 10 in each group), that is, 1—control, 2—PTZ, 3, 4, and 5—treatment groups which received the extract in 25, 50 and 100 mg/kg; i.p, respectively, and 6—positive control group which received valproate (100 mg/kg) as an anticonvulsant drug. All groups were kindled by 11-period injection of PTZ (35 mg/kg i.p.). In the 12th injection, all groups were tested for PTZ challenge dose (75 mg/kg). However, exhibited phase of seizure (0–5) was observed and noted till 30 min after PTZ injection. The results showed that the *F. foetida* gum extract has a reduction effect on seizure intensity, progression, and duration [33].

#### 3.1.11. *Lavandula stoechas*

*Lavandula stoechas* L. (Lamiaceae), known as “Ostokhoddos” in TIM, was mentioned in TIM for epilepsy. The aqueous-methanolic extract of *L. stoechas* flowers was studied for its possible anticonvulsant and antispasmodic activities. When tested in mice, the extract (600 mg/kg) significantly reduced the severity and increased the latency of convulsions induced by PTZ and also reduced PTZ's lethality. *L. stoechas* extract up to a dose of 600 mg/kg was found devoid of any hypnotic effect in mice; however, animals were found to be dull, calm, and relaxed. The sedative effect of the plant extract was confirmed, as it prolonged the pentobarbital sleeping time in mice similar to that of diazepam [36].

#### 3.1.12. *Brassica nigra*

*Brassica nigra *(“Khardal” in TIM) is one of the antiepileptic agents in TIM. In a study by Kiasalari et al., the anticonvulsant effect of *Brassica nigra* was investigated by kindling method. Sixty male mice were divided into six experimental groups (*n* = 10) including 1—control, 2—pentylentetrazole (PTZ)-kindled mice, 3—positive control group received valproate (100 mg/Kg) as an anticonvulsant drug, and 4-5 and 6 received *Brassica nigra* seed extract in three doses (75, 150 and 300 mg/Kg; IP). All groups (except for the control ones) were kindled by 11-period injections of PTZ (35 mg/Kg; IP). In the 12th injection, all groups except for the control group were tested for PTZ challenge dose (75 mg/Kg). However, the exhibited phases of seizure (0–6) were observed and noted till 30 min after the PTZ injection. At last, the brains of all the mice were removed, and then malondialdehyde (MDA), superoxide dismutase (SOD), and nitric oxide (NO) levels of the brain tissues were determined. Statistical analysis of the data shows that the seed extract could reduce the intensity, improvement, and duration of seizure. In addition, the *Brassica nigra* extract increased the SOD and NO levels and decreased the MDA level in the brain tissues. Attained results show that the extract of *Brassica nigra* seed can be used in grand mal seizure treatment. Moreover, the antiepileptic effect of this extract is probably caused by its antioxidant properties and acts via enzyme activity mechanism [34].

#### 3.1.13. *Ruta graveolens*

“Sodab” is a traditional name of *Ruta graveolens* in TIM. It is an another remedy for epilepsy in Iranian traditional books. The anticonvulsant effects of hydroalcoholic extract of rue (*Ruta graveolens*) were investigated in male mice by PTZ-induced seizure. Case groups (five groups) have been injected by 100, 300, 500, 800, and 1000 mg/kg of extracts, positive control group 40 mL/kg phenobarbital and negative control group 10 mL/kg normal saline intraperitoneally. All groups were injected by PTZ (80 mg/kg) intraperitoneally after 45 minutes, and initiation time of myocolnic and tonic-colonic seizures and rate of 24-hour death were measured. hydroalcoholic extracts of rue increased the delay in initiation of myocolonic and tonic-colonic seizures rather than control group dose-dependently and reduced 24-hour seizure induced mortality (*P* < 0.05). Delay in myocolonic seizures in doses of 300, 500, 800, and 1000 mg/kg (*P* < 0.001) and 100 mg/kg (*P* < 0.01) was significant in comparison with negative control group. Delay in tonic-colonic seizures in dose of 1000 mg/kg (*P* < 0.001) and doses of 300, 500, and 800 mg/kg (*P* < 0.05) was significant in comparison with negative control group. So, it seems that the extract of this herb has a decremental effect on PTZ induced seizure in male adult mice [37].

## 4. Conclusion

Epilepsy is one of the most common neurological disorders which affect about 50 million people worldwide [1]. Ineffectiveness of the antiepileptic drugs in nearly 20% of the cases and the serious side effects and chronic toxicity of these drugs lead to the use of complementary and alternative medicine as a new method for treatment of epilepsy.

TIM consists of the sum total of all the knowledge and practices used in diagnosis, prevention, and elimination in Persia from ancient times to present. In this article the antiepileptic herbs of TIM and modern evidence for the efficacy of them were reviewed based on the published studies.

There are some medicinal plants in TIM for the treatment of epilepsy which their anticonvulsant effect have been confirmed by scientific studies in experimental or clinical studies (Table 1). The anticonvulsant effect of some of these medicinal plants like* Anacyclus pyrehrun, Pimpinella anisum, Nigella sativa,* and *Ferula gummosa* was studied in more than one study with different models of seizure. Also for some of these plants like *Nigella sativa* or *Piper longum* the active constituent responsible for the antiepileptic effect was isolated and studied in different models of seizures. Identification and isolation of the active compounds of the other antiepileptic plants could be performed in future studies.

For some of the herbal products used in TIM such as *Pistacia lentiscus* gum (Mastaki), *Bryonia alba* (Fashra), *Ferula persica* (Sakbinaj), *Ecbalium elaterium* (Ghesa-al Hemar), and *Alpinia officinarum* (Kholanjan), there are no or not enough studies to confirm their benefits in epilepsy. It is suggested that an evaluation of the effects of these plants on different models of epilepsy should be performed.

## Figures and Tables

**Table 1:** Anticonvulsant studies of the efficacy of medicinal plants in TIM.

Name of plant	Part used in TIM	Active constituent	Experimental Model of seizures	Clinical trials	Reference
*Terminalia chebula (Combretaceae) *	Fruits	Ethanolic extract of fruits	PTZ, MES, and PC*		[9]
*Anacyclus pyrethrum (compositae) *	Root	Chloroform fraction of root Root ethanolic extract Hydroalcoholic root extract	PTZ, ICES* MES PTZ, MES		[10–13]
*Pimpinella anisum* (Umbelliferae)	Seed	Anise seed oil Methanol seed extract	PTZ, MES PC		[14–17]
*Laurus nobilis* (Lauraceae)	Fruits (seed)	Leaf essential oil	PTZ, MES		[18]
*Origanum majorana* (Labiatae)	Aerial parts	Pet ether, chloroform, acetone, methanol, and aqueous extracts	PTZ, MES		[19]
*Nigella sativa* (Ranunculaceae)	Seed	Aqueous seed extract Seed essential oil	PTZ PTZ	Childhood refractory epilepsy	[20–22]
		Thymoquinone	PTZ, MES	Childhood refractory epilepsy	[23–25]
*Piper longum* (Piperaceae)	Fruits	Aqueous fruit extract	PTZ, strychnine, and 4-aminopyridine		[26]
		Piperine		Uncontrolled epilepsy**	[27]
			Pilocarpine Threshold doses of kainite L-glutamate, N-methyl-D-aspartate guanidinosuccinate		[28, 29]
*Ferula gummosa* *(Apiaceae) *	Gum resin	Fruit essential oil seed acetone extract	PTZ, MES PTZ, MES		[30–32]
*Ferula assa-foetida* (*Apiaceae) *	Gum resin	Gum ethanolic extract	PTZ		[33]
*Brassica nigra* (Brassicaceae)	Fruits	Seed extract	PTZ		[34]
*Caesalpinia bonducella* (Caesalpiniaceae)	Fruits	Seed pet ether extract	PTZ, MES, strychnine, and PC		[35]
*Lavandula stoechas* (Lamiaceae)	Aerial parts	Aqueous-methanolic extract of flower	PTZ		[36]
*Ruta graveolens* (Rutaceae)	Aerial parts	Hydroalcoholic extract	PTZ		[37]

*PTZ: pentylenetetrazole, MES: maximum electroshock, PC: picrotoxin, ICES: increasing current electroshock.

**Effect of piperin on oral bioavailability of phenytoin.
